# Individual and Combined Effects of Diabetes and Glaucoma on Total Macular Thickness and Ganglion Cell Complex Thickness: A Cross-sectional Analysis

**DOI:** 10.18502/jovr.v17i4.12303

**Published:** 2022-11-29

**Authors:** Dhruven Shah, Rita Dhamankar, Vijay Shetty, Suhas S Haldipurkar, Prakash Chipade, Shabnam Tanwar, Prachi Sankhe, Devendra Venkatramani, Paresh Mhatre, Maninder Singh Setia

**Affiliations:** ^1^Laxmi Eye Institute, Maharashtra, India; ^3^Dhruven Shah: https://orcid.org/0000-0001-6683-4161; ^4^Maninder Singh Setia: https://orcid.org/0000-0003-1291-9033

**Keywords:** Glaucoma, Diabetes, Combined Effects, Macular Thickness, Ganglion Cell Complex Thickness

## Abstract

**Purpose:**

Presence of diabetes in glaucoma patients may influence findings while documenting the progression of glaucoma. We conducted the study to compare individual and combined effects of diabetes and glaucoma on macular thickness and ganglion cell complex thickness.

**Methods:**

The present study is a cross-sectional analysis of 172 eyes of 114 individuals. The groups were categorized according to the following conditions: glaucoma, diabetes mellitus, both glaucoma and diabetes (`both' group), and none of these conditions (`none' group). Patients with diabetes did not have diabetic retinopathy (DR). We compared retinal nerve fiber layer (RNFL) thickness, ganglion cell complex (GCC) thickness, foveal loss of volume (FLV), and global loss of volume (GLV) among the groups. We used random effects multivariate analysis to adjust for potential confounders.

**Results:**

The mean (SD) age of these individuals was 60.7 (10.1) years. The total average RNFL and GCC were significantly lower in the glaucoma group (RNFL: -36.27, 95% confidence intervals [CI]: -42.79 to -29.74; P < 0.05, and GCC: -26.24, 95% CI: -31.49 to -20.98; P < 0.05) and the `both' group (RNFL: -24.74, 95% CI: -32.84 to -16.63; P < 0.05, and GCC: -17.92, 95% CI: -24.58 to -11.26; P < 0.05) as compared with the `none' group. There were no significant differences in the average RNFL values and total average GCC between the diabetes group and the `none' group. The values of FLV and GLV were significantly higher in the `glaucoma' group and the `both' group as compared with the `none' group. The foveal values were not significantly different across these four groups. Among the glaucoma cases, 25% were mild, 30% were moderate, and 45% were severe; there was no significant difference in the proportion of severity of glaucoma between the `glaucoma only' and `both' groups (p=0.32). After adjusting for severity and type of glaucoma, there were no statistically significant differences in the values of average RNFL (6.6, 95% CI: -1.9 to 15.2; P=0.13), total average GCC (3.6, -95% CI: -2.4 to 9.6; P=0.24), and GLV (-3.9, 95% CI: -9.5 to 1.6; P=0.16) in the `both group' as compared with the glaucoma only group.

**Conclusion:**

We found that diabetes with no DR did not significantly affect the retinal parameters in patients with glaucoma. Thus, it is less likely that thickness of these parameters will be overestimated in patients with glaucoma who have concurrent diabetes without retinopathy.

##  INTRODUCTION

Glaucoma is characterized by progressive optic neuropathy caused by high intraocular pressure (IOP) leading to gradual death of retinal ganglion cells (RGC). The most common type of glaucoma is primary open angle glaucoma (POAG).^[1,2]^ The inner retinal layers also known as the ganglion cell complex (GCC) are composed of macular nerve fibre layer (NLF), ganglion cell layer (GCL), and inner plexiform layer (IPL), which are specifically involved in glaucomatous damage.^[3]^ Accuracy of GCC measurement in detecting glaucoma is comparable to the detection of glaucoma by measuring peripapillary retinal nerve fibre layer (RNFL) thickness. GCC measurements can potentially be used to monitor glaucoma progression.^[4,5,6]^ Zeimer and colleagues suggested that analyzing macular parameters may be used as an alternative or additional parameter to peripapillary RNFL thickness in diagnosing glaucoma.^[7]^ It has been shown that macular thickness is correlated with optic disc cupping and RNFL thickness in diagnosing glaucoma.^[8,9]^ Furthermore, macular thickness also correlates with RGC counts and perimetry parameters in both glaucomatous and normal eyes when diagnosing glaucoma and analyzing its progression.^[8,9][10][11]^

The estimated global prevalence of diabetes was 9.3% in 2019.^[12]^ Diabetic retinopathy (DR) is the most common microvascular complication of diabetes mellitus. Studies in experimental animal models have indicated that neuroglial tissue loss may occur at early stages of diabetic retinopathy and even precede vascular changes.^[13,14,15]^ In diabetic patients, adenosine monophosphate‑activated protein kinase activation and metabolic stress probably occur as a result of hyperglycemia, hypoglycemia, and hypoxia; thus, the intraretinal neural tissue may not adapt to the metabolic stress of diabetes.^[16,17]^

This may partially explain the pathogenesis of neurodegeneration as an additional component to microvascular pathomechanism of diabetic retinopathy. Araszkiewicz et al suggested that intraretinal neural tissue loss associated with type 1 diabetes directly affects neurodegeneration.^[18]^ Other authors reported that inner retinal layers including RNFL, GCL, IPL in the macula had lesser thickness in patients with type 2 diabetes and early diabetic retinopathy as compared to controls.^[19]^ Furthermore, they also found a linear correlation between GCL thickness and duration of diabetes.^[19]^ Other authors have also found a relationship between reduction and the duration of diabetes, where the reduction in RNFL thickness was associated with longer duration of diabetes and this loss was evident in diabetics with and without diabetic retinopathy.^[20,21]^

Although studies have reported that diabetes is an additional risk factor for POAG, the association is controversial.^[22,24,25]^ Macular thickness measurement (using asymmetry analysis) can help for detection and progression of glaucoma, and is useful in determining the role of diabetes in neurodegeneration.^[26]^ It is quite likely that presence of diabetes in glaucoma patients may influence the findings while documenting the progression of glaucoma. Previous studies have compared the retinal nerve fibre layer in patients with glaucoma and diabetes versus those without diabetes. Some have found no difference in RNFL thickness in diabetic versus non-diabetic POAG patients; whereas other authors have suggested that diabetes may over-estimate the progression of glaucomatous optic neuropathy.^[27,28]^

Thus, we conducted the present study to compare the individual and combined effects of diabetes and glaucoma on macular thickness and ganglion cell complex thickness.

##  METHODS

This cross-sectional analysis was performed on 172 eyes of 114 individuals. The study was conducted at Laxmi Eye Institute, Panvel, India. We recruited the following four types of individuals: those with glaucoma, diabetes mellitus, both glaucoma and diabetes (`both' group), and those without any of these conditions (none group). Patients with glaucoma (primary open angle or angle closure glaucoma) diagnosed by Anderson's criteria (three non-edge points on the pattern deviation map, pattern standard deviation (PSD), and glaucoma hemifield test) were included in the glaucoma group. Individuals who were diagnosed with diabetes (based on fasting blood sugar of ≥ 126 mg/dl, two hour post prandial blood sugar of ≥ 200 mg/dl, or glycated hemoglobin of ≥ 6.5 %)s) were included in the diabetes group and individuals with both diabetes and glaucoma were included in the `both group'. Consenting individuals in the age group of 40-80 years were included for the present study. The exclusion criteria were: presence of any intraocular or neurological diseases affecting RNFL, optic disc, visual field and macular thickness (due to any reason other than diabetes and glaucoma), diabetic patients with DR, past history of any treatment for diabetic retinopathy; past history of any vitreoretinal surgery, prior history of uveitis/retinal disease, significant media opacity (such as corneal scarring, any opacity affecting visual axis, advanced cataract or vitreous opacities which could affect retinal and perimetry scans); hyperopia > +3D; and high myopia > -6D.

All patients underwent complete ophthalmological examination. These were: 1) Best corrected visual acuity (BCVA) for distance and near (using Snellen's Chart and logMAR) and axial length; 2) Measurement of IOP using Goldmann applanation tonometer; 3) Four mirror gonioscopy (indirect gonioscopy using Zeiss Gonio lens); 4) Slit lamp examination; 5) Dilated fundus examination with slit-lamp biomicroscopy and indirect ophthalmoscopy); 6) Visual field testing using Humphrey Field Analyser (Carl Zeiss, Germany, USA); 7) Optical coherence tomography with RTVue (Optovue, USA); 8) RNFL thickness (superior RNFL, inferior RNFL, average RNFL); 9) GCC thickness -total GCC, superior GCC, inferior GCC; 10) Focal loss volume (FLV) and global loss volume (GLV); 11) Full retinal thickness maps. A glycated hemoglobin (HbA1c) test was also performed in these individuals. We classified the glaucoma into three categories based on the mean deviation (MD) values (early defect, moderate defect, and severe defect).^[29]^

We used spectral domain optical coherence tomography (SD-OCT) RTVue-100 (Optovue Inc., Fremont, California, USA) to evaluate the study parameters. The instrument uses an 840 ± 10 nm wavelength illumination source capable of 26000 A-scans/s with a depth resolution of 5 μ. The peripapillary RNFL thickness map was generated from multiple circular scans in an area of 3.45 mm diameter circle with the optic disc as its center. The distance between the internal limiting membrane and the outer edge of the RNFL was estimated to be the RNFL thickness. The GCC complex thickness maps were taken by multiple horizontal and vertical line scans in a region 1mm temporal to the fovea. The GCC thickness for the present study was the combined thickness of the RNFL, ganglion cell layer, and inner plexiform layer. We also assessed the pattern-based parameters: focal loss volume (FLV) and global loss volume (GLV). The FLV was the percentage of the total sum of statistically significant GCC volume loss divided by the GCC map area. The GLV was the percentage of the sum of negative fractional deviation in the entire measurement area.^[30]^ We assessed the retinal macular thickness by using the Retina Map Scan which consisted of multiple horizontal and vertical line scans all centered at the fovea. We only analysed scans with a signal strength of > 65.

### Statistical Analysis

We estimated the mean and standard deviation (SD), and median and interquartile range (IQR) for continuous variables and the proportions for categorical variables. The means across the groups were compared using analysis of variance (ANOVA) with a pair-wise post-hoc comparison with Tukey's correction. The medians were compared using the Kruskal-Wallis test with Dunn's correction. The proportions were compared using the chi square test or Fisher's exact test for low expected cell counts. We used Pearson's correlation co-efficient (*r*) as measure of correlation between two linear variables.

We used random effects linear models for multivariate analysis. The advantages of these models are that they account for both within-individual and between-individual variance. Since in some instances we used both eyes from the same individual, these models were useful.^[31]^ While building these multivariate models, we did not consider each eye as a separate entity but accounted for the fact that two eyes might belong to the same individual.

Data was entered in Ms Excel (© Microsoft, USA) and analysed using Stata Version 15.1 (© StataCorp, College Station, Texas, USA). A p value of < 0.05 was considered statistically significant.

The study was approved by the Institutional Ethics Committee of Laxmi Eye Institute and Charitable Hospital.

**Table 1:** Table showing the demographic and clinical characteristics of 172 eyes, India.


	**None (A)**	**Diabetes (B)**	**Glaucoma (C)**	**Both (D)**	***P*** **-value**
Total eyes	43	43	43	43	
Total individuals	26	28	29	31	
Age: Mean ± SD	53.7 ± 7	61.5 ± 8.9	61.1 ± 10.1	65.4 ± 8.9	< 0.001
Gender			
Male	16 (62)	19 (68)	15 (52)	22 (71)	0.43
Female	10 (38)	9 (32)	14 (48)	9 (29)	
BCDVA (logMAR)			
Median (IQR)	0 (0 to 0.18)	0 (0 to 0.18)	0.18 (0 to 0.18)	0.18 (0 to 0.18)	0.31
HbA1c: Mean ± SD	5.07 ± 0.31	6.10 ± 0.52	5.15 ± 0.34	6.01 ± 0.66	< 0.001
Intraocular pressure (IOP); Mean ± SD	16.91 ± 3.13	16.62 ± 3.65	16.07 ± 4.21	17.16 ± 4.62	0.61
	
	
BCDVA, best corrected distance visual acuity Both indicates patients with both diabetes and glaucoma HbA1c is glycated hemoglobin					

**Table 2:** Table showing the retinal nerve fibre layer (RNFL) values in patients with diabetes, glaucoma, both, and none (43 eyes each).


	**None (A)**	**Diabetes (B)**	**Glaucoma (C)**	**Both (D)**	***P*** **-value**
Average RNFL	104.1 ± 7.2	99.3 ± 10.2	67.5 ± 13.3	78.5 ± 14.5	b,c,d,e,f
Average superior RNFL	104.2 ± 16.5	101.6 ± 11.5	69.2 ± 15.9	82.1 ± 16.6	b,c,d,e,f
Average inferior RNFL	101.7 ± 7.7	97.0 ± 10.5	65.9 ± 13.0	74.9 ± 13.7	b,c,d,e,f
Superior RNFL	127.3 ± 11.7	121.4 ± 16.3	80.0 ± 20.7	95.5 ± 23.1	b,c,d,e,f
Temporal RNFL	78.1 ± 8.9	74.9 ± 10.9	58.9 ± 15.6	64.6 ± 11.3	b,c,d,e
Inferior RNFL	128.5 ± 11.3	120.5 ± 15.0	76.3 ± 16.3	87.2 ± 20.3	b,c,d,e,f
Nasal RNFL	83.2 ± 8.8	80.8 ± 11.9	54.7 ± 12.1	66.7 ± 13.8	b,c,d,e,f
	
	
${}^{a}$ A vs B < 0.05, ${}^{b}$ A vs C < 0.05, ${}^{c}$ A vs D < 0.05**, ** ${}^{d}$ B vs C < 0.05, ${}^{e}$ B vs D < 0.05, ${}^{f}$ C vs D < 0.05 In average superior RNFL the scan shows the RNFL thickness of entire superior hemisphere (180 degrees) so only 2 values present- Average superior and average inferior. On the other hand, in a superior RNL thickness, only superior 90 degrees thickness is calculated. Foveal thickness is central most thickness of diameter 1mm around the fovea Both indicates patients with both diabetes and glaucoma					

**Table 3:** Table showing the foveal parameters in patients with diabetes, glaucoma, both (diabetes and glaucoma), and none (43 eyes each).


	**None (A)**	**Diabetes (B)**	**Glaucoma (C)**	**Both (D)**	***P*** **-value**
Foveal	244.60 ± 31.61	251.11 ± 20.49	236.00 ± 26.68	244.40 ± 19.03	d
Superior hemisphere parafoveal	307.69 ± 20.52	306.41 ± 18.48	287.09 ± 20.30	297 ± 21.69	b,d
Inferior hemisphere parafoveal	307.51 ± 20.78	304.51 ± 19.49	282.09 ± 21.84	290.09 ± 22.75	b,c,d,e
Temporal parafoveal	297.60 ± 22.45	298.00 ± 19.73	273.67 ± 20.42	283.84 ± 21.77	b,c,d,e
Superior parafoveal	311.95 ± 19.67	308.67 ± 18.19	289.18 ± 20.26	298.56 ± 22.19	b,d
Nasal parafoveal	310.81 ± 22.85	309.83 ± 20.54	293.41 ± 22.51	302.48 ± 23.13	b,d
Inferior parafoveal	310.32 ± 20.04	305.30 ± 19.93	281.16 ± 23.05	289.35 ± 23.70	b,c,d,e
Superior hemisphere perifoveal	287.65 ± 19.07	280.02 ± 16.94	261.20 ± 19.32	267.00 ± 17.37	b,c,d,e
Inferior hemisphere perifoveal	279.88 ± 13.22	272.67 ± 17.96	250.37 ± 18.88	254.63 ± 18.39	b,c,d,e
Temporal perifoveal	274.76 ± 14.23	267.79 ± 17.46	247.79 ± 18.37	252.04 ± 17.91	b,c,d,e
Superior perifoveal	285.04 ± 18.93	276.27 ± 17.48	257.83 ± 20.16	263.18 ± 17.46	b,c,d,e
Nasal perifoveal	302.81 ± 20.38	295.95 ± 17.74	274.18 ± 20.42	282.16 ± 19.31	b,c,d,e
Inferior perifoveal	272.79 ± 12.99	265.25 ± 18.56	242.58 ± 19.60	245.79 ± 18.96	b,c,d,e
	
	
${}^{a}$ A vs B < 0.05, ${}^{b}$ A vs C < 0.05, ${}^{c}$ A vs D < 0.05**, ** ${}^{d}$ B vs C < 0.05, ${}^{e}$ B vs D < 0.05, ${}^{f}$ C vs D < 0.05					

**Table 4:** Table showing the estimates and 95% confidence intervals (CI) multivariate analysis for retinal nerve fibre layer (RNFL), ganglion cell complex (GCC), focal loss of volume (FLV), global loss of volume (GLV), and Foveal in 172 eyes.


	**Average RNFL**	**Total Average GCC**	**Focal loss of volume**	**Global loss of volume**	**Foveal**
	**Estimate (95% CI)**	**Estimate (95% CI)**	**Estimate (95% CI)**	**Estimate (95% CI)**	**Estimate (95% CI)**
None	Reference	Reference	Reference	Reference	Reference
Diabetes	-3.4 (-11.4 to 4.7)	-0.8 (-7.3 to 5.8)	-0.03 (-2.05 to 1.99)	1.55 (-3.70 to 6.80)	9.43 (-6.36 to 25.23)
Glaucoma	*-36.3 (-42.8 *to -29.7)*	*-26.2 (-31.5 *to -20.9)*	*6.50 (4.88 *to 8.13)*	*20.98 (16.76 *to 25.21)*	-3.27 (-16.02 to 9.48)
Both	*-24.7 (-32.8 *to -16.6) * ${}^{,a}$	*-17.9 (-24.6 *to -11.3)* ${}^{,a}$	*4.72 (2.66 *to 6.77)*	*14.84 (9.56 *to 20.12)* ${}^{,a}$	1.63 (-14.19 to 17.45)
Age	0.1 (-0.2 to 0.3)	0.1 (-0.1 to 0.3)	-0.01 (-0.07 to 0.05)	-0.08 (-0.24 to 0.08)	0.25 (-0.23 to 0.73)
Gender			
Female	Reference	Reference	Reference	Reference	Reference
Male	-4.1 (-8.8 to 0.6)	*-4.4 (-8.3 *to -0.6)*	0.79 (-0.40 to 1.97)	*3.07 (0 *to 6.13)*	12.70 (3.49 to 21.89)
HbA1c	-0.6 (-5.2 to 3.9)	-1.8 (-5.6 to 1.9)	0.24 (-0.91 to 1.39)	0.60 (-2.37 to 3.57)	-2.25 (-11.16 to 6.4)
IOP	0.2 (-0.3 to 0.7)	-0.3 (-0.8 to 0.1)	0.001 (-0.13 to 0.13)	0.15 (-0.18 to 0.48)	0.11 (-0.81 to 1.03)
Constant	*101.4 (73.3 *to 129.4)*	*111.6 (88.7 *to 134.5)*	0.20 (-6.86 to 7.27)	-0.47 (-18.75 to 17.81)	229.96 (175.15 to 284.76)
Rho	0.76	0.41	0.43	0.65	0.79
	
	
* p < 0.05; ${}^{a}$ The values in glaucoma patients are significantly lower compared with both group Both indicates patients with both diabetes and glaucoma IOP = Intraocular pressure					

**Figure 1:**
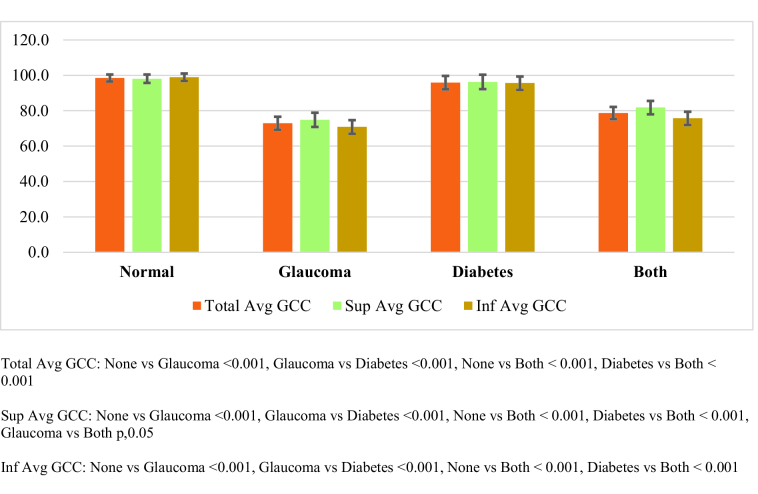
The mean values for ganglion cell complex (GCC) thickness in patients with glaucoma, diabetes, both (diabetes and glaucoma), and none (172 eyes).

**Figure 2:**
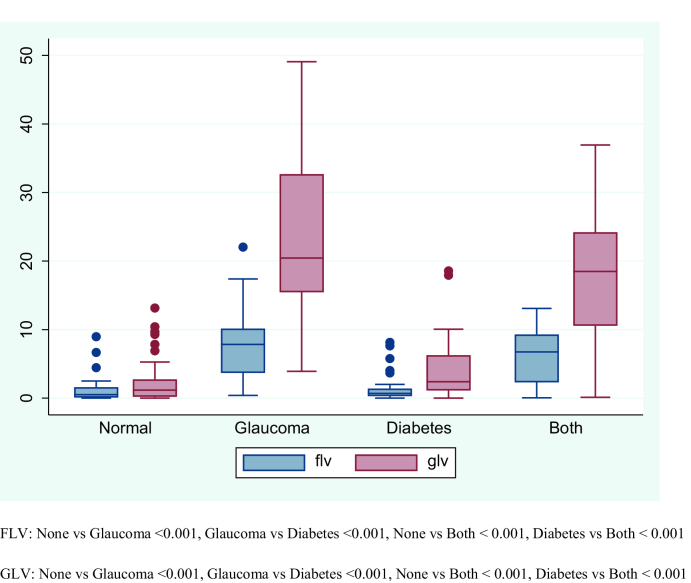
Box plot showing the focal loss of volume (FLV) and global loss of volume (GLV) parameters in patients with glaucoma, diabetes, both (diabetes and glaucoma), and none (172 eyes).

##  RESULTS

The mean ± SD age of participants was 60.7 ± 10.1 years; it was significantly lower in the group without any condition (53.7 ± 7.0) as compared with all the other three groups (Table 1). We included 72 (63%) males and 42 (37%) females in the present study; there was no significant difference in the distribution of gender across these groups (P=0.43). The HbA1C mean ± SD was 5.60 ± 0.67; it was significantly lower in the `none' group (5.07 ± 0.31) as compared with the diabetes group (6.10 ± 0.52) (P < 0.001) and `both group' (6.01 ± 0.66) (P < 0.001). However, there were no significant differences in the HbA1c values between the none group and the glaucoma group (P=0.93) or between the diabetes group and `both group' (P=0.91). The median (IQR, BCVA) in our population was 0.18 (0 to 0.18); it was not significantly different across all four groups. The mean IOP in our population was 16.7 ± 3.9 mm Hg; it was maintained and not significantly different across these groups (P=0.61). We have provided additional details of each parameter in Table 1. The median (IQR, BCVA) duration of diabetes mellitus in our study population was 7 (range, 5 to 12) years. In our glaucoma cases, 25% were mild, 30% were moderate, and 45% were severe; there was no significant difference in the proportion of severity of glaucoma in the `glaucoma only' and `both' groups (P=0.32).

The mean ± SD RNFL thickness was maximum in the `none' (104.9 ± 7.2) group and minimum in the glaucoma group (67.5 ± 13.3). All measurements of RNFL followed this pattern. In general, we found that there was no significant difference in RNFL thickness between the `none' group and diabetes group; however, mean RNFL in the glaucoma and `both' group was significantly lower as compared with the `none' group and diabetes group (Table 2). The mean ± SD total average GCC was also significantly higher in the `none' group as compared with the glaucoma and `both' groups (Figure 1). The median (IQR) FLV in the `none' group (0.51 [0.09 to 1.60]) was significantly lower as compared with the glaucoma (7.84 [3.68 to 10.15]) (P < 0.001) and `both' groups (6.75 [2/31 to 9.29]) (P < 0.001); however, it was not significantly different from the diabetes group (0.70 [0.30 to 1.39]) (P > 0.99). There was no significant difference in the median (IQR) FLV between the glaucoma group and the `both' group (P=0.84) (Figure 2). Similarly, the median (IQR) GLV in the `none' group (1.18 [0.20 to 2.73]) was significantly lower as compared with the glaucoma (20.44 [15.45 to 32.68]) (P < 0.001) or `both' groups (18.49 [10.57 to 24.21]) (P < 0.001); however, it was not significantly different from the diabetes group (2.39 [1.12 to 6.27]) (P=0.33). As with the GLV values, there was no significant difference between the glaucoma group and `both' group (P=0.39) (Figure 2). In general, total foveal value was only significantly different between the diabetes group and glaucoma group. However, individual foveal values were lower in the glaucoma group and `both' group as compared with the `none' group and diabetes group except for superior and nasal parafoveal values which were higher in the glaucoma group when compared to the “both” and “none” groups. The values of these two parameters, i.e. the GLV and FV values were only significantly lower in the glaucoma group as compared with the `none' and diabetes only groups. We have presented all the data in Table 3. Among diabetics, the correlation between HbA1c and average RNFL was weak and not statistically significant (*r* = 0.02; P=0.79); a similar weak correlation was found with individual RNFL values. Similarly, the correlation between HbA1c and total average GCC was weak and not statistically significant (*r* = -0.06; P=0.56); this similar weak correlation was observed in individual GCC values. The mean ± SD axial length (available for analysis) was 23.15 ± 0.99 mm. After adjusting for demographics and type of eye, we found that even though the average RNFL reduced with each mm increase in axial length, it was not statistically significant (Estimate: -5.01, 95% CI: -11.64 to 1.63; P=0.13). Similarly, after adjusting for these parameters, the change in total average GCC was -3.29 units (95% CI: -8,53 to 1.95; P=0.21).

After adjusting for age, gender, HbA1c values, and IOP, we found that the average RNFL and total average GCC were significantly lower in the glaucoma group and `both' group as compared with the `none' group. Furthermore, RNFL and GCC values in the glaucoma group were significantly lower as compared with the `both' group. There was no significant difference in the average RNFL values and total average GCC between the diabetes group and `none' group. The values of FLV and GLV were significantly higher in the `glaucoma' group and `both' group as compared with the `none' group. Only, the GLV was significantly different between the glaucoma group and the `both' group. As with the earlier two parameters (RNFL and GCC), there was no significant difference in the FLV and GLV values between the diabetes group and `none' group. The foveal value was not significantly different across these four groups. In general, age, HbA1c values, and IOP were not significantly associated with any of the parameters. Gender, however, was associated with some of these parameters; males had significantly lower total average GCC values and higher GLV values as compared with females. We have presented all the estimates in Table 4.

In the subgroup analysis (only on patients in the glaucoma or `both' groups), we found that after adjusting for severity (mild, moderate, severe) and type of glaucoma (open angle/close angle) in addition to the above factors, there were no statistically significant differences in the values of average RNFL (6.6, 95% CI: -1.9 to 15.2; P=0.13), total average GCC (3.6, -95% CI: -2.4 to 9.6; P=0.24), and GLV (-3.9, 95% CI: -9.5 to 1.6; P=0.16) in the `both group' when compared with the glaucoma group. The severe glaucoma group however, showed significantly low values of all these parameters. In another subgroup analysis (only patients in the diabetes only or `both' groups), we found that average RNFL (-19.1, 95% CI: -26.1 to -12.1; P < 0.001) and total average GCC (-15.9, 95% CI: -21.6 to 10.3; P < 0.001) were significantly lower in the `both' group as compared with the diabetes only group. Whereas, FLV (4.2, 95% CI: 2.7 to 5.7; P < 0.001) and GLV (11.9, 95% CI: 7.9 to 15.9; P < 0.001) were significantly higher in the `both' group as compared with the diabetes only group. Duration of diabetes was not significantly associated with any of these parameters.

##  DISCUSSION

The study found that average RNFL, average GCC, FLV, and GLV were significantly lower in individuals with glaucoma and those with both glaucoma and diabetes versus the controls or patients with diabetes only. After adjusting for the severity of glaucoma, we did not find any significant differences in the retinal parameters in glaucoma patients with or without diabetes. Furthermore, there were no significant differences in average RNFL, average GCC, FLV, and GLV between controls and diabetes only groups. Thus, we found that the presence of diabetes without retinopathy did not have any significant effect on the retinal parameters either in patients who had glaucoma or those who did not have glaucoma.

It has been hypothesized that degeneration of retinal neurons and glial cells may play a role in the pathogenesis of diabetic retinopathy and may even occur before the development of aneurysms.^[32,33,34]^ Leakage of serum proteins and lipids in the intraretinal space because of increased retinal vascular permeability in diabetic patients may result in higher values of retinal parameters observed in diabetic patients. Araszkiewicz et al found that mean RNFL, and superior and inferior ganglion cell layer (GCL) were significantly thicker in diabetic patients as compared with controls, and were significantly thinner in diabetic patients with retinopathy.^[18]^ A similar finding was also reported by Garcia-Martin and colleagues;^[35]^ who reported that the GCL thickness was significantly less in patients with diabetes as compared with healthy controls. However, in their study, they found that the RNFL was significantly thinner only in the outer inferior quadrant. Another study found that although serum Hb1Ac levels had a significant negative correlation with the whole RNFL thickness, it was not significantly correlated with other parameters such as average macular GCL and average macular thickness.^[36]^ Some authors have suggested that thinning of the inner retina may be seen in patients with diabetes even before changes suggestive of diabetic retinopathy.^[37]^ However, other studies did not find any significant difference in the mean RNFL values between POAG patients with and without diabetes, or correlation between RNFL, and diabetic and ocular parameters.^[27,38]^ As seen in our study, diabetes was not significantly associated with any of these parameters. There were no significant differences between controls and diabetic patients, or between patients who had glaucoma with or without diabetes. Furthermore, neither HbA1c levels nor the duration of diabetes was significantly associated with any of these parameters.

Spaide compared the retinal neurovascular parameters in three groups – controls, diabetic patients, and glaucoma patients.^[39]^ The author found that RNFL thickness and GCL volume were significantly lower in the glaucoma group as compared with the healthy controls. However, only GCL volume was significantly lower in diabetics as compared with healthy controls. Another study reported there was a linear trend of reduction in the RNFL thickness based on the visual field defects in patients with glaucoma.^[40]^ Both of these studies have not used multivariate analysis to estimate the mean differences. In our study, after adjusting for potential confounders (such as age, duration of diabetes, Hb1Ac levels, IOP) we found that there was a significant difference in the retinal parameters between healthy individuals and those with glaucoma, and those with glaucoma and diabetes. However, there were no significant differences between the glaucoma group and those with glaucoma and diabetes. Furthermore, we also found that even though there was a significant reduction in the retinal parameters in individuals with severe VF defects, there was no association with IOP, duration of diabetes, or HbA1c levels. Thus, even though similar pathways of neuroretinal inflammation has been proposed in the retinal changes occurring from both glaucoma and diabetes,^[41,42]^ we did not find the parameters to be significantly altered due to diabetes in individuals with and without glaucoma.

We only assessed data at one point in time– a cross-sectional analysis, so as a consequence a longitudinal follow-up study is recommended to identify the trajectory of the changes to the retinal parameters and its association with progression in glaucoma and diabetes. Nonetheless, as we have used random effects multivariate models to study the association between retinal parameters and the presence of diabetes, glaucoma, or both, this study is an addition to the existing literature. As discussed in the methods section, these models account for both between-individual and within-individual variances.^[31]^ The effect of systemic conditions (such as diabetes) on both eyes in the same individual may be correlated; this needs to be considered while modelling for multivariate analysis. We have used models which account for this correlation. A group of diabetic patients with DR and glaucoma together could potentially show that retinal thickening might hide glaucomatous RNFL and GCC thinning; as a result, we did not include these patients in the study.

In summary, we found that diabetes without retinopathy did not significantly affect the retinal parameters in patients with glaucoma. Furthermore, the values of retinal parameters were not significantly lower in patients with diabetes when compared with healthy controls. We did not find any significant effect of diabetes on RNFL, GCC, and macular thickness parameters. Thus, it is less likely that we may overestimate the thickness of these parameters in patients with glaucoma who have concurrent diabetes without retinopathy.

## References

[B1] Peters D, Bengtsson B, Heijl A (2013). Lifetime risk of blindness in open-angle glaucoma. Am J Ophthalmol Oct.

[B2] Quaranta L, Riva I, Gerardi C, Oddone F, Floriani I, Konstas AG, Adv Ther Jun 2016;33(6):959-81.

[B3] Scuderi G, Fragiotta S, Scuderi L, Iodice CM, Perdicchi A (2020). Ganglion Cell Complex Analysis in Glaucoma Patients: What Can It Tell Us? Eye Brain.

[B4] Lee KH, Kang MG, Lim H, Kim CY, Kim NR (2012). A formula to predict spectral domain optical coherence tomography (OCT) retinal nerve fiber layer measurements based on time domain OCT measurements. Korean J Ophthalmol Oct.

[B5] Gonzalez-Garcia AO, Vizzeri G, Bowd C, Medeiros FA, Zangwill LM, Weinreb RN, Am J Ophthalmol Jun 2009;147(6):1067-74, 1074 e1.

[B6] Wojtkowski M, Srinivasan V, Fujimoto JG, et al (2005). Three-dimensional retinal imaging with high-speed ultrahigh-resolution optical coherence tomography. Ophthalmology Oct.

[B7] Zeimer R, Asrani S, Zou S, Quigley H, Jampel H, A pilot study Ophthalmology. Feb 1998;105(2):224-31.

[B8] Oli A, Joshi D (2015). Can ganglion cell complex assessment on cirrus HD OCT aid in detection of early glaucoma? Saudi J Ophthalmol. Jul-Sep.

[B9] Asrani S, Challa P, Herndon L, Lee P, Stinnett S, Allingham RR, J Glaucoma Apr 2003;12(2):119-28.

[B10] Araie M, Saito H, Tomidokoro A, Murata H, Iwase A, vest Ophthalmol Vis Sci Oct 9 2014;55(11):7199-205.

[B11] Mathers K, Rosdahl JA, Asrani S, J Glaucoma Feb 2014;23(2):e98-104.

[B12] Saeedi P, Petersohn I, Salpea P, et al, Diabetes Res Clin Pract Nov 2019;157:107843.

[B13] Martin PM, Roon P, Van Ells TK, Ganapathy V, Smith SB, vest Ophthalmol Vis Sci Sep 2004;45(9):3330-6.

[B14] Barber AJ, Prog Neuropsychopharmacol Biol Psychiatry Apr 2003;27(2):283-90.

[B15] Cabrera DeBuc D, Somfai GM (2010). Early detection of retinal thickness changes in diabetes using Optical Coherence Tomography. Med Sci Monit Mar.

[B16] Antonetti DA, Barber AJ, Bronson SK, et al, Diabetes Sep 2006;55(9):2401-11.

[B17] Hardie DG, Hawley SA, Scott JW, J Physiol Jul 1 2006;574(Pt 1):7-15.

[B18] Araszkiewicz A, Zozulinska-Ziolkiewicz D, Meller M, et al (2012). Neurodegeneration of the retina in type 1 diabetic patients. Pol Arch Med Wewn.

[B19] van Dijk HW, Verbraak FD, Kok PH, et al, vest Ophthalmol Vis Sci May 14 2012;53(6):2715-9.

[B20] Lee MW, Lim HB, Kim MS, et al, Sci Rep Mar 24 2021;11(1):6813.

[B21] Lim HB, Shin YI, Lee MW, Park GS, Kim JY, JAMA Ophthalmol Oct 1 2019;137(10):1125-1132.

[B22] de Voogd S, Ikram MK, Wolfs RC, et al (2006). Is diabetes mellitus a risk factor for open-angle glaucoma? The Rotterdam Study. Ophthalmology Oct.

[B23] Pasquale LR, Kang JH, Manson JE, Willett WC, Rosner BA, Hankinson SE (2006). Prospective study of type 2 diabetes mellitus and risk of primary open-angle glaucoma in women. Ophthalmology Jul.

[B24] Chopra V, Varma R, Francis BA, et al, Ophthalmology Feb 2008;115(2):227-232 e1.

[B25] Leske MC, Wu SY, Hennis A, Honkanen R, Nemesure B, Group BES (2008). Risk factors for incident open-angle glaucoma: the Barbados Eye Studies. Ophthalmology Jan.

[B26] van Dijk HW, Verbraak FD, Kok PH, et al, vest Ophthalmol Vis Sci Jul 2010;51(7):3660-5.

[B27] Akkaya S, Can E, Ozturk F, t Ophthalmol Oct 2016;36(5):727-36.

[B28] Takahashi H, Goto T, Shoji T, Tanito M, Park M, Chihara E (2006). Diabetes-associated retinal nerve fiber damage evaluated with scanning laser polarimetry. Am J Ophthalmol Jul.

[B29] Susanna R, Staging glaucoma patient: why and how? Open Ophthalmol J Sep 17 2009;3:59-64.

[B30] Arintawati P, Sone T, Akita T, Tanaka J, Kiuchi Y, J Glaucoma Dec 2013;22(9):713-8.

[B31] Snijders TAB, Bosker RJ

[B32] Gardner TW, Antonetti DA, Barber AJ, LaNoue KF, Levison SW, Surv Ophthalmol Dec 2002;47 Suppl 2:S253-62.

[B33] Park SH, Park JW, Park SJ, et al, Diabetologia Sep 2003;46(9):1260-8.

[B34] Peng PH, Lin HS, Lin S, Can J Ophthalmol Aug 2009;44(4):417-22.

[B35] Garcia-Martin E, Cipres M, Melchor I, et al, J Ophthalmol 2019;2019:1825819.

[B36] Gundogan FC, Akay F, Uzun S, Yolcu U, Cagiltay E, Toyran S, Ophthalmologica 2016;235(3):125-32.

[B37] Chhablani J, Sharma A, Goud A, et al, vest Ophthalmol Vis Sci Oct 2015;56(11):6333-8.

[B38] Takis A, Alonistiotis D, Panagiotidis D, Ioannou N, Papaconstantinou D, Theodossiadis P, Clin Ophthalmol 2014;8:455-63.

[B39] Spaide RF (2019). Measurable Aspects of the Retinal Neurovascular Unit in Diabetes, Glaucoma, and Controls. Am J Ophthalmol Nov.

[B40] Geng W, Wang D, Han J, J Ophthalmol 2020;2020:4874876.

[B41] Rubsam A, Parikh S, Fort PE

[B42] Vohra R, Tsai JC, Kolko M (2013). The role of inflammation in the pathogenesis of glaucoma. Surv Ophthalmol Jul-Aug.

